# Promoting Health Equity in an Era of Growing Contradictions Between Capital Accumulation and Social Reproduction in Capitalist Economies

**DOI:** 10.1177/2752535X251370927

**Published:** 2025-08-26

**Authors:** Dennis Raphael, Toba Bryant, Rozhin Amin

**Affiliations:** 1 School of Health Policy and Management, 7991 York University, Toronto, ON, Canada; 2 Faculty of Health Sciences, Ontario Tech University, Oshawa, ON, Canada; 3 PhD Program in Health Policy and Equity, 7991 York University, Toronto, ON, Canada

**Keywords:** health equity, political economy, capital accumulation, social reproduction, post-capitalist futures

## Abstract

**Background:** Despite a robust literature on the importance of promoting health equity, actual progress in doing so is flagging and this is especially the case in Anglo-Saxon liberal welfare states such as Canada, the USA, and the United Kingdom.

**Purpose:** In this paper we place these developments within the context of the political economy concepts of capital accumulation -- or profit making -- and social reproduction -- or the ongoing functioning of society.

**Research Design: **We carefully reviewed the current state of theorization and research into the political economy of health to identify the main themes and findings in this literature in relation to the polycrisis of living and working conditions in Canada, the USA, and United Kingdom.

**Analysis:** We drew upon critical materialist political economy thought to show how the growing contradictions between profit making and societal functioning in capitalist economies – and this especially so in liberal welfare states -- threaten both the quality and equitable distribution of the living and working conditions that shape health – the social determinants of health – and the organization and delivery of health care.

**Results:** While there is increasing application of political economy approaches to understanding the adverse effects of capitalism, almost all of these are limited to critiquing capitalism without envisioning a post-capitalist society. Various ways of addressing these contradictions are provided that include 1) redistribution, social spending by governments, and managing the market economy within existing economic and political structures; 2) movement towards social democratic or conservative models of governance common to the Nordic and Continental nations respectively; or 3) building a post-capitalist socialist future.

**Conclusion: **While we offer three paths forward towards achieving health equity, we conclude that the last path, building a post-capitalist socialist future, offers the most useful means of promoting health equity in both the short and long-term.

Why don’t the gods appear on our markets

And share out, smiling, nature’s plenty

And allow the people, fortified by food and wine

To be good to one another henceforth, and friendly?^1^

-- Bertolt Brecht, 1941/2019

## Introduction

Equity in health is about everyone attaining their full health potential by not experiencing avoidable circumstances that would prevent this.^2^ Promoting health equity through the enactment of public policy that equitably distributes the social determinants of health and provides access to health care is a dominant theme in public health literature across the globe.^3^ Achieving such equity has become increasingly difficult in this era of neoliberalism and this is especially the case in nations identified as liberal welfare states such as Canada, the United States of America (USA), and the United Kingdom (UK)^4–7^ Neoliberalism is problematic for health as it advocates free market capitalism involving retrenchment of the welfare state, privatization of public institutions, imposition of austerity, and deregulation of business practices, all identified as threatening health.^4^

There are different approaches to understanding how public policies are developed and implemented which are relevant to achieving health equity.^8,9^ These include pluralist approaches to policy change that assume that transmitting ideas and research findings to governing authorities will lead to progressive change. Another approach posits that institutionalist analyses of governmental structures that shape the making of public policy will facilitate authorities’ responsiveness to change. Finally, there are a variety of political economy models that offer insights into how economics and politics inform public policymaking that shape the distribution of resources necessary for health and the organization and delivery of health care.

We argue that even then, the scope of many of these political economy models is limited to critique of existing circumstances and fail to offer an alternative to the capitalist economic system that is responsible for the lack of health equity. These pluralist, institutionalist and political economy approaches have not been particularly successful in achieving health equity in the liberal welfare states of Canada, USA, and UK where the market, rather than the state, is the dominant societal institution.^4,10–12^

In this article we argue that achieving health equity requires recognition of the inherent contradictions -- common to all forms of capitalist welfare states – between the core requirement of capitalist economies of capital accumulation or profit making and social reproduction or the ongoing functioning of society.^13,14^ We see these contradictions as having become so great in Canada, the USA and UK as to have created a polycrisis in the quality and distribution of the social determinants of health and the organization and delivery of health care. Amidst this polycrisis, promoting health equity is difficult, if not impossible.^15,16^

We provide three paths forward for promoting health equity in these nations: (1) increasing redistribution and social spending by governments and managing the market economy within existing economic and political structures; (2) moving towards social democratic or conservative models of governance common to the Nordic social democratic and Continental conservative nations respectively; or (3) building a post-capitalist socialist future. Of these three paths, we argue that the third – movement towards a post-capitalist socialist society – is the preferred way forward. A substantial body of literature in the critical materialist stream of political economy (defined below) literature supports this view and is making its way into mainstream health equity discourse.

## Promoting Health Equity

Whitehead’s landmark work on health equity of 35 years ago still best defines the concept of health equity and the means of promoting it: “Equity in health implies that ideally everyone should have a fair opportunity to attain their full health potential and, more pragmatically, that no one should be disadvantaged from achieving this potential, if it can be avoided.”^2^ ^(p.67)^ For Whitehead there are two key components of health equity: equity in access to the living and working conditions necessary for health and equity in access to required health care. Barriers to equity are usually a result of systemic societal structures and processes.

Regarding access to the conditions necessary for health, the absence of health equity is the presence of health inequalities, which, since most are avoidable and unfair, are actually health inequities. Whitehead outlines four processes that create these inequities: (1) health-damaging behaviour where the degree of choice of lifestyles is severely restricted; (2) exposure to unhealthy, stressful living and working conditions; (3) inadequate access to essential health and other public services; and (4) health-related social mobility whereby sick people move down the social scale.

Whitehead identifies seven means of addressing health inequities which clearly involve the making and implementation of public policy: (1) improving living and working conditions; (2) enabling people to adopt healthier lifestyles; (3) committing to decentralizing power and decision making, thereby encouraging people to participate in every stage of the policy-making process; (4) assessing the health impacts of policies and implementing health-equity supportive ones through intersectoral action; (5) concern and control at the international level; (6) making high quality health care accessible to all; and (7) assuring equity policies based on appropriate research, monitoring and evaluation.

Thousands of works since then have provided a myriad of proposals for achieving health equity based on these concepts. The report of the World Health Organization’s Commission on Social Determinants of Health’s Closing the Gap in a Generation: Health Equity Through Action on the Social Determinants of Health is a core document offering these requirements: (1) improve daily living conditions; (2) tackle the inequitable distribution of power, money and resources; and (3) measure and understand the problem and access the impact of action.^17^ ^(p. 2)^ More recent health equity work is documented in Bryant’s *Handbook of the Social Determinants of Health*;^18^ for Canada in Raphael’s *Social Determinants of Health: Canadian Perspectives*;^19^ the USA in Bezruchka’s *Inequality Kills Us* *All*;^20^; and the UK in Walsh and McCartney’s *Social Murder? Austerity and Life Expectancy in the UK*.^21^ The World Health Organization’s follow-up to its 2008 report, *World Report on Social Determinants of Health Equity, 2025*, is now available.^2^

None of these make for happy reading. Increases in income and wealth inequality, employment insecurity and precarity, and housing and food insecurity have led to a polycrisis of living and working conditions in many nations -- and this is especially the case in the liberal nations of Canada, USA and UK – where the ongoing functioning of society is uncertain.^13,14,22–24^ Like others, we argue this failure is due in part to health equity researchers and advocates neglecting how the distribution of power and influence among societal sectors create the inequitable distribution of resources necessary for health and problematic aspects of the organization and delivery of health care.^25–28^

However, we also argue that even when unequal power and influence are considered in these works, there is frequently an unwillingness to acknowledge how these differences are integral features of the capitalist economic system and are unlikely to be remedied unless this system is radically transformed or replaced.^29^ This unwillingness is rooted in the dominant models researchers and advocates use to understand public policy change that preclude consideration of radical transformations in existing economic and political systems.^30–34^

The models used to promote health equity used by many researchers and advocates adhere to the pluralist view that quality research informed by quality theory leads to progressive public policy.^8,9^ For example, pluralism implies that all who wish to influence public policy have the ability to do so and that governments will respond to evidence. This is a misguided and naïve view of the public policy process in which government is perceived as a benevolent ruler that carefully weighs evidence and makes decisions believed to be best for the whole of society. In short, pluralism has an underdeveloped understanding of inequality and its causes.

Others subscribe to the institutionalist view that by understanding processes of government policymaking, health equity-promoting public policy will be implemented through research and advocacy.^8,9^ Institutionalism is another rational public policy model that tends to be primarily concerned with the knowledge activities of upper-level policy analysts within government and those associated with think-tanks. It also provides a rather limited understanding of how the public policy process works. In short both pluralism and institutionalism tend to depoliticize the public policy process and how it can create and perpetuate social and health inequalities. Neither model considers the dominance of some groups such as the corporate and business sector and their considerable capacity to influence public policy decisions to protect its own class interests. Both models therefore, neglect issues of class relations.

We believe that in liberal welfare states where the dominant societal institution is the market, the public policy playing field is skewed in favor of the wealthy and powerful such that these models lack usefulness. This is because research and advocacy efforts often cannot overcome the power and influence of the corporate and business sector whose profitmaking would not be served by equalizing the distribution of economic and other resources.^4^ Political economy models of public policymaking are more likely to address these larger issues.

## Political Economy of Health

Political economy of health approaches consider health and health care as shaped by the relationships between individuals and societies, markets and the state, and economic and political systems.^35,36^ The origins of political economy are in the writings of seventeenth- and eighteenth-century philosophers James Stuart, Jean-Baptiste Colvert, Adam Smith, David Hume, and Francois Quesnay.^37^ These works emphasize the role of the market over the state in promoting well-being with the best example being Adam Smith’s famous notion of the ‘invisible hand,’ whereby state policies are less effective in advancing social welfare than the self-interested market exchanges of individuals.^38^ These market-oriented ideas dominate contemporary governing authorities’ and public discourses on the role of markets and states in producing and distributing resources in capitalist societies. These dominant approaches do not consider how the class interests of the business and corporate sector take precedence over those of workers or those unable to work because of lack of jobs, illness, disability, or discrimination.

Considering this neglect of class issues, it is surprising that many in the health equity field usually assume that the “political economy of health” is concerned with addressing imbalances in power and influence that block the promotion of health equity.^36,39^ But as noted by Harvey:

When political economy is invoked in the [public health] literature, it is not always explicitly defined. In those instances when it is defined, no standard definition is evident. This is especially problematic because various theoretical traditions that employ the term “political economy” -- such as Keynesian, neo-classical, neoliberal, institutional, rational choice, and Marxian—approach questions of political economy in often widely discrepant ways.^40^ ^(p. 294)^

We employ the stream of political economy identified by David Coburn as critical materialist political economy (CMPE).^41^ As a political economy approach CMPE is concerned with economics and politics. CMPE is materialist in that it sees societal structures and processes as shaped by material relations of power and influence organized along class lines. Therefore, resource production and distribution – including the social determinants of health and health care -- emanate from these relations. A materialist analysis also sees the ideas a society and its members hold about the nature of society, health, and health care as shaped by these same material conditions.^42,43^

CMPE is critical in that it posits that people within society may not have accurate understanding of how their society actually works. If they do not, CMPE aims to shift these views such that they can now ask how things could be different. Thus, CMPE examines how the material conditions of life – the economic base of society -- shapes the political structures and processes of a society and the dominant ideas – the superstructure – common to a society.^44^ CMPE has proven useful in explorations of the quality and distribution of the social determinants of health,^45–47^ the sources of food insecurity,^48^ women’s employment,^49^ children’s health and well-being,^50^ policymakers’ perspectives on health equity,^51^ the role of polemic in public policy change,^52^ the commodification of knowledge work,^53^ the organization of call centres in India,^54^ and the environmental crisis.^55^

Within CMPE, two key mechanisms are capital accumulation -- or profit making -- and social reproduction -- or the wellbeing of society. When out of balance, they produce contradictions that makes the achievement of health equity difficult, if not impossible. We see the contemporary form of capitalism – neoliberal global capitalism – as producing contradictions of such magnitude that a polycrisis of both health determinants and health care now exists.

One example of the value of a CMPE approach – although not so named at that time -- is seen as early as 1979 in Doyal and Pennell’s volume The Political Economy of Health which examined in detail the adverse effects of capitalism upon health and the need to replace it:

It follows from this that the demand for health is in itself a revolutionary demand and one which must be taken seriously in the broader struggle for socialism in both developed countries and the third world.^56^ ^(p. 297)^

Many CMPE concepts such as the health impacts of neoliberalism;^57–61^ how corporate power and influence shape health;^62–68^ the commercial determinants of health;^69–76^ financialization;^77,78^ and even the adverse health effects of capitalism^79–87^ are now part of the health equity literature.

However, unlike Doyal and Pennell,^56^ most of these analyses are limited to critiquing capitalism and proposing means of managing its adverse effects. There is infrequent analyses of how these structures and processes are integral to capitalist economies such that what may be required for achieving health equity is radical transformation or replacement of the capitalist economic system. Indeed, frequently works on health equity and the social determinants of health often do not even mention capitalism or socialism or do so in passing.^88–98^ Why is this the case? In the words of primary care physician, historian, and sociologist Howard Waitzkin:

Most of us find that it is difficult to imagine a viable path from capitalism to post-capitalism (the ‘TINA’ perspective, that is, “There Is No Alternative”). Because it is hard to imagine a viable path from capitalism to post-capitalism, most people addressing our world’s challenges assume that capitalism will continue to exist. Therefore, we engage in peculiar ways of struggling to improve our most important problems without confronting capitalism, even though we recognize that capitalism generates these problems and continues to make them worse.^99^ ^(p. 1)^

## The Elephant in the Room: Capitalism

The Merriam-Webster Dictionary defines the expression “elephant in the room” as “an obvious major problem or issue that people avoid discussing or acknowledging.”^100^ For us, the elephant in the health equity room is the contemporary form of neoliberal global capitalism. We are not alone in this belief. Not only is there an extensive political science literature of the catastrophic effects of capitalism on all aspects of contemporary life,^24,101–108^ there is an increasing willingness in the mainstream public health and health care community to acknowledge capitalism as a primary threat to health equity through its adverse effects upon access to food, education, housing, income and wealth, quality employment, and transportation.^40,81,109–112^ How does capitalism threaten health equity?

Capitalism requires that society be organized along capital accumulation by the production and then selling of products at values greater than what is involved in producing them, i.e., raw materials, infrastructure such as buildings and machinery, and wages.^113,114^ These processes of production and distribution usually produce profound social inequalities which must be legitimized since Braudel notes that “Capitalism, the privilege of the few, is unthinkable without the active participation of society.”^115^ ^(p. 50)^ Without such legitimization and consent of those being governed, discontent can escalate into organized resistance. Ideas of individual freedom, equality of opportunity, and the virtues of free enterprise provide such legitimization.

Wright considers the problems of capitalism – clearly related to adverse health effects – within three clusters: Equality/fairness, democracy/freedom, and community/solidarity.^112^ Regarding equality/fairness, Wright argues: “In a just society, all persons would have broadly equal access to the material and social means necessary to live a flourishing life.”^116^ ^(p. 10)^ By any measure, capitalism makes the attainment of fairness in terms of life outcomes difficult for many. While the structures, processes, and effects of capitalism were managed during the period of monopoly capitalism from 1945 to 1975, under contemporary neoliberal global capitalism (see below) the current era sees social inequalities increasing with day-to-day life for most not improving or worsening. Neoliberal governance has led to growing corporate domination of the political process such that the state is reduced to the role of handmaiden of this sector.

For Wright, democracy and freedom are linked and support self-determination: “In a fully democratic society, all people should have broadly equal access to the necessary means to participate meaningfully in decisions about things that affect their lives.”^116^ ^(p. 15)^ Doing so requires access to power and the ability to shape public policy that affect their lives.

Global neoliberalism has the corporate and business gaining greater control over public policy that organizes production and distribution of economic and other resources.^107,117^ The corporate and business sector also comes to dominate societal discourse concerning the nature of economic and political processes -- justifying these changes -- through its control of the mainstream media and through the action of corporate and business-friendly political parties.^114^ Democracy comes to be reduced to voting for one of the pro-business parties that dominate public discourse.^118,119^ The illusory process of freedom comes to mean little since as Coburn notes “capitalists preach democracy, but practise power.”,^41^ ^(p. 68)^

Wright states: “Community/solidarity expresses the principle that people ought to cooperate with each other not simply because of what they personally get out of it, but also out of a real commitment to the wellbeing of others and a sense of moral obligation that it is the right thing to do.,^116^ ^(p. 18)^ Such a communitarian view of society provides a sense of purpose and meaning in life and supports equality/fairness and democracy/freedom.

The lack of democratic processes and the growing competition for resources amongst the population under capitalism weakens already fragile concepts of community and solidarity. Neoliberal processes – now dominant in this age of global capitalism -- celebrate the fraying of ties amongst the population thereby reducing the sense of common purpose and weakening the public’s ability to control their future.^120,121^

A core concept related to capitalism’s effects upon health is exploitation. We think about exploitation in two ways. The first is Wright’s concept of economic oppression.^122^ This occurs when (1) the material welfare of one group is causally related to the material deprivations of another; (2) the causal relation in (1) involves coercively enforced exclusion from access to productive resources; and (3) this exclusion is morally indictable. In this definition, deprivation is caused by excessive accumulation by others. The second is that exploitation is an inherent feature of capitalism. For Marx, the worker forced during employment to produce surplus value to benefit those who control the means of production is capitalism’s key feature of exploitation.^123^ Another aspect of capitalism is the alienation of workers who are estranged from the products of their labour and lack the power to control the production and distribution of economic and other resources. Øversveen and Kelly and Baum use alienation theory to explain the adverse health effects of income inequality in capitalist societies.^124,125^ Dawson raises the question of whether alienation is part and parcel of capitalism and therefore present for all or only afflicts those being left out.^126^

We see the current polycrisis of income and wealth inequalities, employment insecurity and precarity, and housing and food insecurity resulting from excessive capital accumulation at the expense of social reproduction fitting into Wright’s concept of economic oppression. The following section considers the concepts of capital accumulation and social reproduction in greater detail.

## Capital Accumulation and Social Reproduction

Though 35 years old, we find compelling and useful Ross and Trachte locating the evolution of capitalism into three distinct eras: entrepreneurial capitalism (through most of the 19^th^ century), monopoly capitalism (through most of the 20^th^ century), and global capitalism (from 1975 onwards).^127^ For them – and us -- the post-1975 era of global capitalism emphasizes the free movement of capital across borders, with multinational corporations operating on a global scale and influencing global trade, production, and labour markets. Ross and Trachte’s view of this phase as dominated by corporate interests, where the demands of the working class are marginalized, public expenditures on supports and services are cut back, and there is stagnating purchasing power of the working class rings true for us today.

During late monopoly capitalism (from 1945 to 1975) -- the “Golden Age of Capitalism -- conditions of work became more regulated, and through collective organization, workers curbed employer power and negotiated better wages and working conditions. Additionally, governments assumed responsibility for welfare services to ensure their equitable distribution. However managed capitalism struggled during the 1970s allowing entry of a new perspective – neoliberalism – that promoted the revival of market forces embodied in government policies of economic globalization, trade liberalization, scaling back of the welfare state, and privatization of publicly held institutions. This new phase of global capitalism brought about declines in union strength, increased job insecurity, and growing income and wealth inequalities. Its effects are now seen as the polycrisis of rising income and wealth inequalities, precarious employment, housing unaffordability, growing food insecurity, and declining access to healthcare.^29^

Ross and Trachte’s analysis proved profoundly prescient. Teeple^34^ draws similar conclusions as to the effects of neoliberal inspired governance corresponding to their concept of global capitalism as do Labonte and Stuckler.^58^ Hallstrom specifies the post-1975 period as implicated in the profound shift in public policymaking in Canada away from redistribution and social spending.^128^ As a result the welfare state is now ineffective in balancing capital accumulation and social reproduction, and this is especially so in liberal welfare states.^4,29^

## Contradictions and the Contemporary Polycrisis

The concept of polycrisis characterizes an array of linked crises that are not simply contemporaneous but also interconnected. Many consider the polycrisis as incorporating ecology, democracy, digitalization as well as economic challenges.^129–131^ In our work we focus on the skewing of living and working conditions – or social determinants of health – and see all of these and the broader issues noted above as growing from the ongoing contradictions of contemporary neoliberal global capitalism. In such a situation, conventional public policy management methods are ineffective as the polycrisis extends beyond any single policy area and reflects the overall structures and processes of a society’s political economy.^14^ We see the polycrisis as driven by private sector need for capital accumulation which threatens social reproduction. The importance of capital accumulation to health is not a novel insight: Doyal and Pennell identified capital accumulation as being the core of why governing authorities frequently fail to meet the health and health care needs of their citizens.^56^ The contemporary polycrisis is now finally directing public health attention to its effects.

The depth of the social determinants of health polycrisis is seen in the similarities of health threatening Victorian-era capitalist practices of the mid-1850s and contemporary Canadian society in income and wealth inequalities, employment security and working conditions, and access to food, housing, and health services, deaths of despair in the USA, and declining life expectancy in the UK.^21,80,132^ In Canada, the relentless quest for capital accumulation has resulted in precarious work and stagnating wages for many as the profits of the companies they work for grow, housing unaffordability as private realty companies buy up currently affordable housing and carry out renovictions and raise rents, and increasing food insecurity as the major food distributors record growing profits.^19^ Similar processes are taking place in the USA and UK.^133,134^

The second component of health equity is access to health care. Health care has always been a site for private sector capital accumulation through investment and growing commodification in Canada and other countries with public health care systems.^56,82,135^ Commodification through private for-profit investment undermines access to health care with special impact on the health of those in the lowest income groups.^136^ Health care became commodified as governments shifted away from the post-World War II commitment to provide health care and public health to their populations.^34^

Countries vary in their investments in these programs, especially health care. The USA is the only wealthy country without a public health care system yet consistently outspends all other rich nations on health.^137^ USA health care is costly as a result of amalgamation of hospitals and providers in which the growth of private monopolies exact higher prices from insurance payers.^82^ This leads to adverse financial and health impacts for users. For example, a 2016 study found that cancer treatment can result in bankruptcies and increased the risk of mortality from treatable cancers.^138^

Growing private sector involvement is apparent in Canada and the UK public health care systems with adverse consequences for health.^139–143^ These impacts reflect the impact of commodification and contribute to a growing health care crisis as countries with public health care systems privatize health services as a cost-saving measure which may lead to preventable illness and deaths, increasing infant mortality and declining life expectancy, some of which have already occurred.

## Analysis: Emerging Themes in the Political Economy of Health and Health Care

We see the critical stream within the political economy of health literature as increasingly focused upon neoliberal capitalism and how it makes achieving health equity difficult. In our reading of the literature we see four main themes: (1) the adverse health effects of neoliberal capitalism and the creation of the polycrisis; (2) increasing corporate and business domination of the public policy agenda; (3) the rise of right-wing populist parties in response to deteriorating living and working conditions; and (4) increasing privatization of public health care.

## Recognizing the Adverse Health Effects of Neoliberal Global Capitalism

It is rather striking how the last few years have seen an increase in literature linking features of capitalism to problematic quality and distributions of the social determinants of health^79,80,83,85,142^ and health care.^82,84^ McNamara and Bambra for example, map out the polycrisis and its relation to health inequalities.^16^ While they mention the failure of free market capitalism to deliver its promised effects, they do not explicitly call for radically reforming or replacing capitalism itself, a common failure, as we noted earlier, to most working in the political economy of health area. Instead, Bambra, Lynch, and Smith, as example, show how progressive policy implementation promoted health during “The Great Society” in the USA, England’s “Health Inequalities Strategy”, and “German Reunification.”^57^ Whether such innovations are possible in our current period of neoliberal global capitalism is uncertain.^107^

The earliest and perhaps still the best exposition of the adverse effects of unrestrained capitalism is Friedrich Engels’s *Condition of the Working Class in England*.^144^ Medvedyuk, Govender and Raphael document how Engels’s description of the adverse effects of capitalism as social murder was virtually absent for 150 years from any academic discourse but has seen a striking revival in the public health literature.^145^ This has been driven by events such as the Grenfell Fire in London UK, the imposition of austerity in many nations, and the differential health effects of the COVID-19 epidemic.

Additionally, Govender, Medvedyuk, and Raphael document the increasing framing of these adverse health effects as social murder by the mainstream media.^146^ In the UK the health effects of imposition of austerity have been termed social murder^21^ and in the USA, the term has been used to describe the effects of corporate authoritarianism overtaking the democratic rule of law.^147^ Another thread of literature describes these adverse effects on those occupying specific social locations. Gender, race, and Indigeneity interact with class-related capitalist structures to cause adverse health outcomes for women, racialized, and Indigenous populations.^148,149^ The role capitalism played, and still plays, in colonialism falls within this thread.^150–152^

It is important to note that even when the term social murder is applied to contemporary issues such as the Grenfell Fire, austerity in the UK, and the effects of the COVID-19 epidemic, these works do not necessarily implicate capitalist exploitation as being behind it.^145,153^ In many cases, the cause is simply considered to be bad public policy, illustrating the resistance to tackling capitalism head on, and calling for its radical transformation or replacement.

## Increasing Corporate and Business Domination of the Public Policy Agenda

Two threads in the public health literature focus on corporate and business influences upon health equity-related public policy. The first falls under the heading of the commercial determinants of health and focuses on how profit making drives the production and marketing of health threatening food and drink.^76,154,155^ The second focuses on corporate and business influences upon the quality and distribution of social determinants of health of income, housing and food, governmental supports, and health care.^62–70,156,157^

Holden and Lee detail two ways corporate power shapes public policy. Structural power is when governments have no choice but to safeguard the needs of business due to the ability of corporations to move elsewhere.^158^ This may include reducing taxes on these corporations and the wealthy, reducing social spending, limiting the ability of workers to unionize, weakening legislation on employment security and working conditions, including wages and benefits, facilitating financialization of the housing market and food industries by large corporate interests and privatizing previous public institutions such as electricity, transportation, and health care. All of these serve to increase capital accumulation, but do at the expense of social reproduction, as indicated by declining quality and equitable distribution of the social determinants of health and access to health care.

When structural power is insufficient to achieve their goals, corporate interests turn to agency approaches of political engagement through direct lobbying and providing gifts and funds to politicians, as well as indirect lobbying and political activities (for example, via the creation and support of think tanks, consultancies and research institutes).^159,160^ Institutional participation (for example, with business people holding key positions on government commissions and boards of hospitals and schools) provides a further avenue for influence.^156^

## The Rise of Right-Wing Populist Parties in Response to Deteriorating Conditions

Another feature of neoliberal capitalism with adverse effects upon the objective of achieving health equity is the rise of right-wing populism in many nations including Canada, USA and the UK and several European nations.^161,162^ It is associated with the rise of anti-immigrant sentiment, distrust of government and endorsement of authoritarian leaders as manifested in the USA, Hungary, Poland, and even the Nordic nations.^163^

Rinaldi and Bekker argue that right-wing populist parties threaten progressive health policy by calling for restricting access and eligibility to welfare programs for marginalized immigrants and minority groups.^164^ Since welfare generosity is related to population health outcomes, the rise in strength of right-wing populist parties is likely to adversely affect the achievement of health equity. Similar conclusions are reached by Stronks and Agyemang who direct focus to discrimination in interpersonal relationships and weakened public support for equity-oriented health policies.^165^

The rise of right-wing populist parties has been attributed to the deteriorating living and working conditions in the current polycrisis of neoliberal capitalism and to the failure of politicians and leaders of the left to provide an alternative vision of society.^161,162,166^ While social democratic parties were initially organized to seek improvements in living and working conditions through parliamentary democracy with the ultimate goal of a post-capitalist socialist society, most – and this is especially the case in liberal welfare states -- eschewed this latter hope in favor of managed reforms within a capitalist economy.^167^ More recently, these parties have embraced neoliberal approaches to governance providing little distance between them and traditional pro-capitalist political parties.^168,169^ Przeworski terms these successive phases of social democracy as revolution, revisionism, remedialism, and resignation which now offers few means of countering the neoliberal resurgence now adversely affecting people’s lives.^170^ What such alternative visions would look like are provided in later sections.

## Increasing Privatization of Public Health Care

As noted, jurisdictions with public health care systems increasingly privatize health care services.^139–142,171^ They justify privatization as saving taxpayers’ money, but said taxpayers end up paying out of pocket for health care. Right-wing populists have seized upon the anger of the working class to promote privatization that ultimately serve the interests of the corporate and business sector at the expense of workers.^172,173^ Privatization surged following the COVID-19 pandemic as public health care systems were stretched to meet needs as the virus spread. This is especially the case in liberal welfare states such as Canada and UK, among others.^174,175^ The adverse effects of privatization on health and health care are well documented.^138–141,176–178^

## The Ways Forward

We see three paths forward towards health equity for liberal welfare states in the health equity literature: (1) Reforms within existing economic and political structures; (2) Moving towards social democratic and conservative forms of the welfare state; and (3) Building a post-capitalist socialist future.

## Reform: Addressing Taboos of Redistribution, Social Spending, and Managing the Market Economy

Lynch presents three public policy taboos among contemporary policymakers that make achieving health equity by reducing health inequalities difficult: redistribution, social spending, and managing the market economy.^179^ These taboos are important in liberal welfare states as indicators of redistribution, social spending and managing the market economy fall well below all social democratic welfare states and most conservative ones.^4^ Many health equity researchers and advocates work for reforms within existing systems with remarkably consistent pleas for public policy action.^180^

Regarding redistribution, proposals include increasing progressivity of the tax structure and promoting universal programs linked to greater social spending on family benefits, pharmacare and denticare, job training, pensions and social assistance and disability benefits.^180^ For managing the market economy, workplace legislation and regulation related to employment security, working conditions, and wages and benefits are required. Nations can enable the unionization of workplaces and the extent of collective bargaining through sectoral and intersectoral employment agreements which also serves to manage the market economy. Nations can legislate employment security by managing lay-offs and terms of part-time and temporary employment. Liberal nations provides little of this.^181^

## Moving towards Social Democratic and Conservative Models of Governance

Esping-Andersen places the structures and processes of resource provision within different forms of the capitalist welfare state.^182,183^ Three forms – social democratic, conservative, and liberal – differ in how and the extent to which they provide economic and social security. Key aspects of these forms of the welfare state are the extent of stratification, decommodification, and the mix of state, market, and family resource provision. Note that the terms liberal and conservative in the welfare state literature have a very different meaning from their common usage in American politics. Liberal welfare states are the least developed and the conservative and social democratic welfare states are more developed with many similarities.^184^

Liberal welfare states provide the least economic and social security. Liberal welfare states (e.g., Canada, the UK and USA) provide modest benefits that the state provides when the market fails to meet citizens’ most basic needs Their political and social history is one of dominance by business interests resulting in the population relying on the employment marketplace rather than the state as the source of economic and social security. A key feature is use of means-tested benefits and lower social expenditures accompanied by generally lower tax rates.

The social democratic welfare states (for example, Finland, Sweden, Denmark and Norway) emphasize universal welfare rights and provide generous benefits and entitlements. Their political and social history is one of political dominance by social democratic parties of the left, a result of political organization initiated by industrial workers and farmers that later came to include the middle class.^185^ Through universal provision of a range of benefits, these states have historically secured the loyalties of a significant proportion of the population. The strong influence of organized labour contributes to the stability of the social democratic welfare state by moderating the influence of the business sector on public policymaking. Their key feature of this welfare state is greater social expenditures – possible through generally higher taxation rates – that provide universal benefits across the life-course.

The conservative welfare state (for example, Belgium, France, Germany, the Netherlands and Switzerland) also offers generous benefits but does so through social insurance plans based on employment status.^182,183^ Emphasis is on supporting the primary wage earner, usually male. Their political and social history is one of political dominance by Christian Democratic parties where traditional Church concerns with maintaining the family merges with conservative upholding of status differences among citizens. These tendencies sometimes manifest in corporatist approaches (for example, Germany) where business interests are major influences or Statist approaches (for example, France) where the State plays a key role in provision of citizen security.^186^

Bryant and Raphael^4^ among others^185^ show how stratification is greater and decommodification -- the ability to live a decent life outside of having paid employment -- is lower in liberal welfare states such that the quality and equitable distribution of the social determinants of health in social democratic welfare states are clearly superior to what is seen in liberal welfare states and somewhat better than those of conservative welfare states. This is so since liberal welfare states have the market as its central institution and its ideological inspiration is one of limiting state interventions in the market economy.^12^ As a result, the health-enhancing features of collective employment bargaining coverage and universal benefits of the social democratic and conservative welfare states are for the most part absent in liberal welfare states.

How apparent are the effects of these differences upon societal health and well-being? In one analysis, a Society index made up of measures of poverty, income inequality, intergenerational income mobility, gender wage gap, immigrant wage gap, racial wage gap, income of people with disabilities, voter turnout, jobless youth, life satisfaction, social network support, homicides, burglaries, and suicides, finds the social democratic nations of Norway, Denmark, Sweden, and Finland ranking 1st, 2nd, 3rd, and 5th, while Canada ranks 13 of 26 comparison nations with the UK ranked 17th, and USA 26th^187^ On an index of Social Justice for Organization for Economic Cooperation and Development nations consisting of measures of poverty, education, the labor market, intergenerational justice, health, and social inclusion and nondiscrimination, Norway, Denmark, Sweden, and Finland score 2-5 (Iceland scores 1) while the liberal nations score lower: UK (11th), Canada (12th), and USA (36st).^188^

We see two dominant critiques of this welfare state approach in the literature. The first is concern with the validity of the classification of nations into these types. While there is certainly valid debate concerning the placement of nations such as Belgium, Switzerland and the Netherlands, there is good evidence for the validity of the liberal and social democratic welfare states. Of the 12 welfare state typologies identified by Bambra, in six of the seven that have included Canada, Canada is found in the group similar to the so-called liberal welfare state: liberal, basic security, or liberal Anglo-Saxon.^189^ The USA is so placed in 8 of 8 and the UK in 7 of 10. The Nordic nations are placed consistently in the social democratic, encompassing, Nordic, or services groups: Finland (8 of 11), Denmark (9 of 11), Sweden (12 of 12), and Norway (12 of 12).

The second issue is the presence of differences in health among the different forms of the welfare state. Reviews usually find that social democratic welfare states produce smaller absolute health inequalities and better overall population health.^6,190–194^

Muntaner and colleagues find that social democratic regimes fare best on absolute health outcomes.^195^ McCartney et al. and Bambra conclude, respectively:

Social democratic welfare states, higher public spending, fair trade policies, extensions to compulsory education provision, improved access to health care, and high-quality affordable housing have positive impacts on population health… Several authors have found that Nordic welfare states tend to have lower overall mortality rates than do other European types, but greater health inequalities. However, this is not the case if inequalities are measured via life span variation.^196^ ^(p. e1-2)^

Social-democratic welfare states, which had the lowest overall levels of social inequality, also tended to have better health outcomes for those at the top and those at the bottom of the socioeconomic ladder: more equal countries almost always do better in social and health outcomes.. And despite the fact that some of the liberal welfare states, like the UK and Canada, had robust, well-funded universal healthcare systems, these countries joined the other members of the liberal world in having especially large and intractable health inequalities, resulting from large and intractable socioeconomic inequalities.”^134^ ^(p. 81)^

Liberal nations perform especially poorly in what is usually seen as a sensitive measure of population health, infant mortality rates. The social democratic welfare states of Finland, Norway, and Sweden are ranked in the top five of 36 OECD nations with Denmark being just out of the top 10 with a rank of 11. The liberal welfare states of Canada, UK, and USA are all ranked 25^th^ or below with Canada (30th) and the USA (32nd) doing especially poorly.^197^

Berkowitz^198,199^ provides elegant arguments for moving towards such forms of governance as do others.^5,7,57^ What would be needed to move towards alternatives to liberal models of governance? Raphael shows how the distinctive social democratic and conservative welfare states have developed due to central state rather than federal governance, significant left-party support, high union densities in the social democratic welfare states and high employer-supported collective agreement rates in conservative nations, and proportional representation in the electoral process rather than the first-past-the-post system.^200^ In regards to a left political party, the USA does not have one, and the erstwhile social democratic parties of Canada – the New Democratic Party – and UK – Labour -- have long abandoned any critique of neoliberal capitalism^166^ making movement towards alternative forms of welfare states unlikely. Additionally, the embrace of neoliberal approaches to governance across all nations suggest that even these alternative forms of the welfare states are not sustainable, requiring envisioning post-capitalist social futures.^201–208^

## Building a Post-capitalist Socialist Future

What might such a society look like? McBride calls for a “radical transformation” whereby popular sovereignty comes to control capital; rebuilding of the public domain and state; and socialization of capital investment.^107^ A new regime would respect human rights and create a state where meeting people’s needs is primary. Carroll calls for a “green socialism” that moves not only from fossil capitalism but also from capitalism itself by bringing under public control, energy, water and other utilities, expanding public sector services, adopting principles of economic democracy, redistributing wealth, and socializing investment.^209^

Freudenberg outlines how health and public health workers and others can “build a movement for a better world.”^81^ Advocacy positions would include calling for an expanded public sector; strengthening democracy; confronting systemic racism and sexism; transforming the discussion about taxes and regulations; and making science and technology public property through popular education, political mobilization, nationalization of major industries, and erosion of capitalist institutions by strengthening alternative democratic structures such as credit unions. Raphael and Bryant bring together recent works on moving towards a post-capitalist socialist future.^29^

## Conclusion

In this paper we have shown how concepts from the political economy of health literature illuminate how capitalist structures and processes adversely affect health. We have also detailed the apparent reluctance of many of the contributors in this field to call for the radical transformation or replacement of this problematic economic system.^1^

We do not have such reluctance but recognize, as does McBride, that reform within existing economic and political structures would provide some short-term but limited improvements.^107^ Moving liberal political economies such as Canada, the USA, and UK towards the arguably superior governance models common in the social democratic and conservative nations would promote health equity. Yet, there is now a literature about the inability of even these welfare states to manage the contradictions between capital accumulation and social reproduction.^210– 213^ Even when managing capital accumulation within a nation, the adverse effects of Norwegian capital accumulation, as one example, on other nations are ignored.^214,215^

We cannot be sure of what a post-capitalist socialist society will look like. Yet, the well-documented adverse health effects of neoliberal global capitalism require that we nevertheless move towards that goal.^216^ For Harvey, we have no option, as the inherent contradictions of capitalism will lead to its demise.^217^ Kovel argues that these contradictions are leading to the end of a livable planet.^218^ We agree with Meiville, who in a discussion of the impending climate catastrophe – with relevance for the polycrisis in social determinants of health and health care -- states: “Socialism, wherein the astonishing scientific and technical powers of humanity are harnessed to need, for all the uncertainties and errors that would occur, would give an infinitely greater likelihood of sustaining a habitable world than more of the same system that got us here.”^215,218,219^ ^(p. 161)^

## Data Availability

All data used in this study are from publications provided in the reference section.*

## Note

1 Beyond the immediate scope of our paper yet relevant to promoting health equity are Wright’s six means of moving beyond capitalism: smashing capitalism, dismantling capitalism, taming capitalism, resisting capitalism, escaping capitalism, and Wright's preferred option and eroding capitalism.^116^ We favor the electoral approach provided earlier. Flanagan and Raphael explore how credit unions could begin a transition to a post-capitalist society by providing a model of member-controlled non-profit institutions focused on community wellbeing rather than profit making.^219^

## References

[1] Brecht B . Song of the defencelessness of the gods and the good from the good person of Szechwan. In: Kuhn T (ed). Constantine (translators) The collected poems of Bertolt Brecht. W.W. Norton and Company, 1941/2019.

[2] Whitehead M . The concepts and principles of equity and health. World Health Organization, Regional Office for Europe, 1990.

[3] World Health Organization . World report on social determinants of health equity. 2025. https://www.who.int/publications/i/item/9789240107588

[4] Bryant T Raphael D . The politics of health in the Canadian welfare state. Canadian Scholars’ Press, 2020.

[5] Côté D Raynauilt MF . Scandinavian common sense: policies to tackle social inequalities in health. Baraka Books, 2015.

[6] Heins E Dukelow F . Liberal welfare states. In: Greve B (ed). De Gruyter Handbook of contemporary welfare states. De Gruyter, 2022, pp. 85–100.

[7] Olsen GM . Poverty and austerity amid prosperity: a comparative introduction. University of Toronto Press, 2021.

[8] Bryant T . Parameters of public policy change. Ijcyfs 2015; 6(2): 295–307.

[9] Bryant T . Health policy in Canada. Canadian Scholars’ Press, 2016.

[10] Borras AM . A critical political economy of health inequities. In: The Routledge Handbook of the political economy of health and healthcare. Routledge, 2024, pp. 119–130.

[11] Raphael D . Introducing the politics of food insecurity. In: Mendly-Zambo Z Raphael D (eds). The politics of food insecurity in Canada and the United Kingdom. Policy Press, 2025, pp. 1–33.

[12] Saint-Arnaud S Bernard P . Convergence or resilience? A hierarchial cluster analysis of the welfare regimes in advanced countries. Curr Sociol 2003; 51(5): 499–527.

[13] Jayasuriya K . Polycrisis or crises of capitalist social reproduction. Glob Soc Chall J 2023; 2: 203–211.

[14] Penner M . The paradox of polycrisis: capitalism, history, and the present. Journal Hist 2023; 58(2-3): 152–166.

[15] Khosla R Sen G Ghebreyesus TA , et al. Many crises, one call to action: advancing gender equality in health in response to polycrises. Lancet 2024; 404(10454): 731–733. 39067459 10.1016/S0140-6736(24)01450-8

[16] McNamara C Bambra C . The global polycrisis and health inequalities. Int J Soc Determinants Health Health Serv 2025; 27551938251317472. 10.1177/27551938251317472 PMC12171050 39957184

[17] World Health Organization . Closing the gap in a generation: health equity through action on the social determinants of health. 2008. https://www.who.int/publications/i/item/WHO-IER-CSDH-08.1 10.1016/S0140-6736(08)61690-6 18994664

[18] Bryant T (ed). Handbook on the social determinants of health. Edward Elgar Publishing, 2025.

[19] Raphael D . Social determinants of health: Canadian perspectives. Canadian Scholars’ Press, 2025.

[20] Bezruchka S . Inequality kills us all: COVID-19’s health lessons for the world. Taylor & Francis, 2022.

[21] Walsh D McCartney G . Social murder? Austerity and life expectancy in the UK. Policy Press, 2024.

[22] Albert MJ . Capitalism, complexity, and polycrisis: toward neo-Gramscian polycrisis analysis. Glob Sustain 2025; 8: e7.

[23] Dinan S Beland D Howlett M . How useful is the concept of polycrisis? Lessons from the development of the Canada emergency response benefit during the COVID-19 pandemic. Policy Design and Practice 2024; 7: 430–441.

[24] Szepanski A . Capitalism in the age of catastrophe: the newest developments of financial capital in times of polycrisis. Springer Nature, 2024.

[25] Friel S Marmot M . Global health inequities: structures, power, and the social distribution of health. In: Routledge handbook of global public health. Routledge, 2010, pp. 65–79.

[26] Friel S Arthur M Frank N . Power and the planetary health equity crisis. Lancet 2022; 400(10358): 1085–1087. 35964591 10.1016/S0140-6736(22)01544-6

[27] Friel S Townsend B Fisher M , et al. Power and the people's health. Soc Sci Med 2021; 282: 114173. 34192622 10.1016/j.socscimed.2021.114173

[28] Harris P Baum F Friel S , et al. A glossary of theories for understanding power and policy for health equity. J Epidemiol Community Health 2020; 74(6): 548–552. 32198290 10.1136/jech-2019-213692

[29] Raphael D Bryant T . Socialism as the way forward: updating a discourse analysis of the social determinants of health. Crit Public Health 2023; 33(4): 387–394.

[30] Das RJ . Marx’s Capital, capitalism and limits to the state: theoretical considerations. Routledge, 2022.

[31] Das RJ . Capital, capitalism and health. Crit Sociol 2023; 49(3): 395–414.

[32] Das R . Understanding Marx on health: toward a class-based approach. In: The Routledge handbook of the political economy of health and healthcare. Routledge, 2024, pp. 47–59.

[33] Das RJ Latham RE Fasenfest D (2025). Being a critical social scientist. Class, race and corporate power, 13:(1), Article 3.

[34] Teeple G . Globalization and the decline of social reform: into the twenty-first century. Garamond Press, 2000.

[35] Mooney G . The health of nations: towards a new political economy. Bloomsbury Publishing, 2012.

[36] Primrose D Loeppky R . Revitalizing the political economy of health and healthcare in a context of crisis. In: The Routledge handbook of the political economy of health and healthcare. Taylor & Francis, 2024.

[37] Whiteside H . Capitalist political economy: thinkers and theories. Routledge, 2020.

[38] Smith A . An inquiry into the nature and causes of the wealth of nations. An Electronic Classics Series Publication, 2005. https://www.rrojasdatabank.info/Wealth-Nations.pdf

[39] Mantoura P Morrison V . Policy approaches to reducing health inequalities. Montreal: National Collaborating Centre for Health Public Policy, 2016. https://nccdh.ca/resources/entry/policy-approaches-to-reducing-health-inequalities

[40] Harvey M . The political economy of health: revisiting its Marxian origins to address 21st-century health inequalities. Am J Publ Health 2021; 111(2): 293–300. 10.2105/AJPH.2020.305996 PMC7811101 33351658

[41] Coburn D . Health and health care: a political economy perspective. In: Bryant T Raphael D Rioux M (eds). Staying alive: Critical perspectives on health, illness, and health care. 2nd ed. Toronto: Canadian Scholars’ Press, 2010, pp. 65–92.

[42] Medvedyuk S Raphael D . Defining health through a critical materialist political economy lens. Glob Health Promot 2024; 31(2): 34–42. 37823385 10.1177/17579759231194600 PMC11363467

[43] Medvedyuk S Raphael D . Promoting social justice in the capitalist academy? Health equity and the Johns Hopkins university Michael bloomberg school of public health. Capital & Class, 2024, p. 03098168241234304.

[44] Vidal M Adler P Delbridge R . When organization studies turns to societal problems: the contribution of Marxist grand theory. Organ Stud 2015; 36(4): 405–422.

[45] Bryant T . Introduction to the handbook on the social determinants of health. In: Handbook on the social determinants of health. Edward Elgar Publishing, 2025, pp. 2–11.

[46] Borras AM Mohamed FA . Health inequities and the shifting paradigms of food security, food insecurity, and food sovereignty. Int J Health Serv 2020; 50(3): 299–313. 32200687 10.1177/0020731420913184

[47] Mendly-Zambo Z . Food insecurity in Canada and the United Kingdom. In: Mendly-Zambo Z Raphael D (eds). The Politics of Food Insecurity in Canada and the United Kingdom. Policy Press, 2025, pp. 34–71.

[48] Mendly-Zambo Z Raphael D . Competing discourses of household food insecurity in Canada. Soc Pol Soc 2019; 18(4): 535–554.

[49] Raphael D . Welfare states, cultural values and women’s precarious work: insights from welfare state and cultural values theory. Crit Stud 2018; 14(1): 46–60.

[50] Raphael D . The parameters of children’s health: key concepts from the political economy of health literature. Ijcyfs 2015; 6(2): 186–203.

[51] Komakech MDC (2022). From the standpoint of policymakers: an institutional ethnography inquiry into the policymaking process to address health equity and social determinants of health in Canada. [PhD dissertation]. York University.

[52] Raphael D Bryant T Govender P , et al. Desperately seeking reductions in health inequalities in Canada: polemics and anger mobilization as the way forward? Sociol Health Illness 2022; 44(1): 130–146. 10.1111/1467-9566.13399 34741772

[53] Popowich S . ChatGPT and the commodification of knowledge work. CAUT Journal, 2024.

[54] Roy A . Spaces of labour process: a case study of call centres in Kolkata, India. Ottawa: Library and Archives Canada Bibliothèque et Archives Canada, 2007.

[55] Flanagan E . Climate change action versus complacency: how political power and influence shape national environmental policy. Major Research Paper, York University, 2022.

[56] Doyal L Pennell I . The political economy of health. Pluto Press, 1979.

[57] Bambra C Lynch J Smith KE . Getting better: the policy and politics of reducing health inequalities. Policy Press, 2025.

[58] Labonté R Stuckler D . The rise of neoliberalism: how bad economics imperils health and what to do about it. J Epidemiol Community 2016; 70(3): 312–318. 10.1136/jech-2015-206295 26424847

[59] Mooney G . Neoliberalism is bad for our health. Int J Health Serv 2012; 42(3): 383–401. 22993960 10.2190/HS.42.3.b

[60] Schrecker T . Neoliberalism and health: the linkages and the dangers. Sociology Compass 2016; 10(10): 952–971.

[61] Schrecker T Bambra C . How politics makes us sick: neoliberal epidemics. Springer, 2015.

[62] Burgess R Nyhan K Freudenberg N , et al. Corporate activities that influence population health: a scoping review and qualitative synthesis to develop the HEALTH-CORP typology. Glob Health 2024; 20(1): 77. 10.1186/s12992-024-01082-4 PMC11549802 39516852

[63] Freudenberg N Galea S . The impact of corporate practices on health: implications for health policy. J Publ Health Pol 2008; 29: 86–105. 10.1057/palgrave.jphp.3200158 18368021

[64] Lacy-Nichols J Marten R Crosbie E , et al. The public health playbook: ideas for challenging the corporate playbook. Lancet Glob Health 2022; 10(7): e1067–e1072. 35623376 10.1016/S2214-109X(22)00185-1 PMC9197808

[65] Madureira Lima J Galea S . Corporate practices and health: a framework and mechanisms. Glob Health 2018; 14: 21. 10.1186/s12992-018-0336-y PMC5815179 29448968

[66] Mialon M Vandevijvere S Carriedo-Lutzenkirchen A , et al. Mechanisms for addressing and managing the influence of corporations on public health policy, research and practice: a scoping review. BMJ Open 2020; 10(7): e034082. 10.1136/bmjopen-2019-034082 PMC7371213 32690498

[68] Ulucanlar S Lauber K Fabbri A , et al. Corporate political activity: taxonomies and model of corporate influence on public policy. Int J Health Pol Manag 2023; 12: 7292. 10.34172/ijhpm.2023.7292 PMC10462073 37579378

[69] Wiist WH (ed). The bottom line or public health: Tactics corporations use to influence health and health policy, and what we can do to counter them. Oxford University Press, 2010.

[70] McKee M Stuckler D . Revisiting the corporate and commercial determinants of health. Am J Publ Health 2018; 108(9): 1167–1170. 10.2105/AJPH.2018.304510 PMC6085040 30024808

[71] Gilmore AB Fabbri A Baum F , et al. Defining and conceptualising the commercial determinants of health. Lancet 2023; 401(10383): 1194–1213. 36966782 10.1016/S0140-6736(23)00013-2

[72] Friel S Collin J Daube M , et al. Commercial determinants of health: future directions. Lancet 2023; 401(10383): 1229–1240. 36966784 10.1016/S0140-6736(23)00011-9

[73] Freudenberg N Lee K Buse K , et al. Defining priorities for action and research on the commercial determinants of health: a conceptual review. Am J Publ Health 2021; 111(12): 2202–2211. 10.2105/AJPH.2021.306491 PMC8667845 34878875

[74] Maani N Petticrew M Galea S (eds). The commercial determinants of health. Oxford University Press, 2023.

[75] Kickbusch I Holly L . Digital governance needed to tackle commercial determinants of health. Lancet 2024; 403(10438): 1747. 10.1016/S0140-6736(23)02283-3 38704162

[76] Kickbusch I Allen L Franz C . The commercial determinants of health. Lancet Glob Health 2016; 4(12): e895–e896. 27855860 10.1016/S2214-109X(16)30217-0

[77] Friel S Schram A Frank N , et al. Financialisation: a 21st century commercial determinant of health equity. Lancet Public Health 2024; 9(9): e705–e708. 39214638 10.1016/S2468-2667(24)00187-7

[78] Hunter BM Murray SF . Deconstructing the financialization of healthcare. Dev Change 2019; 50(5): 1263–1287.

[79] Edwards N King J . Neoliberal capitalism and its impact on individual and community health. In: Edwards N King J (eds). Social work practice in health: An introduction to contexts, theories and skills. Taylor & Francis, 2022, pp. 25–36.

[80] Case A Deaton A . Deaths of despair and the future of capitalism. Princeton University Press, 2021.

[81] Freudenberg N . At what cost: modern capitalism and the future of health. Oxford University Press, 2021.

[82] Christiansen I . Commodified healthcare, profits, priorities and the disregard for life under capitalism. In: Primrose D Loeppky D Chang R (eds). The Routledge handbook of the political economy of health and healthcare. Routledge, 2024, pp. 223–233.

[83] Minkler M Wallace SP McDonald M . The political economy of health: a useful theoretical tool for health education practice. Int Q Community Health Educ 1994; 15(2): 111–126. 20841021 10.2190/T1Y0-8ARU-RL96-LPDU

[84] Perry T Bernasek A . Profits over care? An analysis of the relationship between corporate capitalism in the healthcare industry and cancer mortality in the United States. Soc Sci Med 2024; 349: 116851. 38642520 10.1016/j.socscimed.2024.116851

[85] Balfe M . Key sociological concepts for medicine: capitalism and health. J R Soc Med 2024; 117(3): 96–99. 38656900 10.1177/01410768241245591 PMC11145303

[86] Noonan RJ . Capitalism, health and wellbeing: rethinking economic growth for a healthier, sustainable future. Emerald Publishing Limited, 2024.

[87] Noonan RJ . Extrinsic goals benefit capitalism but not well-being. Rethinking the economy’s goal for a healthier future. Health Promot Int 2024; 39(5): daae090. 39322425 10.1093/heapro/daae090 PMC11424164

[88] Gómez CA Kleinman DV Pronk N , et al. Addressing health equity and social determinants of health through healthy people 2030. J Publ Health Manag Pract 2021; 27(Supplement 6): S249–S257. 10.1097/PHH.0000000000001297 PMC8478299 33729197

[89] Heller JC Givens ML Johnson SP , et al. Keeping it political and powerful: defining the structural determinants of health. Milbank Q 2024; 102(2): 351–366. 38363858 10.1111/1468-0009.12695 PMC11176401

[90] Hiam L Klaber B Sowemimo A , et al. NHS and the whole of society must act on social determinants of health for a healthier future. BMJ 2024; 385: e079389. 38604669 10.1136/bmj-2024-079389

[91] Brown TH Homan P . The future of social determinants of health: looking upstream to structural drivers. Milbank Q 2023; 101(Suppl 1): 36–60. 37096627 10.1111/1468-0009.12641 PMC10126983

[92] Chelak K Chakole S . The role of social determinants of health in promoting health equality: a narrative review. Cureus 2023; 15(1): e33425. 36751221 10.7759/cureus.33425 PMC9899154

[93] Krieger N . Theories for social epidemiology in the 21st century: an ecosocial perspective. Int J Epidemiol 2001; 30(4): 668–677. 11511581 10.1093/ije/30.4.668

[94] Krieger N . Got theory? On the 21st century rise of explicit use of epidemiologic theories of disease distribution: a review and ecosocial analysis. Curr Epidemiol Rep 2014; 1: 45–56.

[95] Labonté R . Ensuring global health equity in a post-pandemic economy. Int J Health Pol Manag 2022; 11(8): 1246. 10.34172/ijhpm.2022.7212 PMC9808344 35942959

[96] Lynch J . The political economy of health: bringing political science in. Annu Rev Polit Sci 2023; 26(1): 389–410.

[97] McCartney G Popay J . Are place-based approaches to reducing health inequalities a highway to success or a policy dead-end? The Journal of Critical Public Health 2025; 1246–1250.

[98] McCartney G Collins C Mackenzie M . What (or who) causes health inequalities: theories, evidence and implications? Health Policy 2013; 113(3): 221–227. 23810172 10.1016/j.healthpol.2013.05.021

[99] Waitzkin H . “Post”-pandemic capitalism: reform or transform? Comment on “Ensuring global health equity in a post-pandemic economy”. Int J Health Pol Manag 2023; 12: 7936. 10.34172/ijhpm.2023.7936 PMC10425694 37579389

[100] Merriam-Webster Dictionary . Elephant in the room. 2025. https://www.merriam-webster.com/dictionary/elephantintheroom

[101] Callinicos A . The new age of catastrophe. John Wiley & Sons, 2023.

[102] Cairó-i-Céspedes G Castells-Quintana D . Dimensions of the current systemic crisis: capitalism in short circuit? Prog Dev Stud 2016; 16(1): 1–23.

[103] Pavlov EV . The current crisis and the cost of capitalism. Radic Philos Rev 2010; 13(2): 215–221.

[104] Robinson WI . Global capitalism and the crisis of humanity. Cambridge University Press, 2014.

[105] Sassoon D . Morbid symptoms: an anatomy of a world in crisis. Verso Books, 2021.

[106] Smith ME Butovsky J Watterton JJ . Twilight capitalism: Karl Marx and the decay of the profit system. Fernwood Publishing, 2021.

[107] McBride S . Escaping dystopia: rebuilding a public domain. Policy Press, 2022.

[108] Klein N . This changes everything: capitalism vs. the climate. Simon & Schuster, 2014.

[109] Benach J Pericàs JM Martínez-Herrera E , et al. Public health and inequities under capitalism: systemic effects and human rights. In: Philosophical and methodological debates in public health. Springer International Publishing, 2019, pp. 163–179.

[110] Levins R . Is capitalism a disease? In: Hofrichter R (ed). Health and social justice: Politics, ideology, and inequity in the distribution of disease. Jossey Bass, 2003, pp. 365–384.

[111] Raphael D Bryant T . Crisis of capitalism: social welfare states or socialist states as the way forward? In: Bryant T (ed). The handbook of the social determinants of health. Edward Elgar, 2025, pp. 371–384.

[112] Sell SK Williams OD . Health under capitalism: a global political economy of structural pathogenesis. Rev Int Polit Econ 2020; 27(1): 1–25.

[113] Hammer D . Capitalism explained. 2022. https://www.wealthsimple.com/en-ca/learn/capitalism-explained

[114] Fulcher J . Capitalism: a very short introduction. (2nd edition). Oxford University Press, 2015.

[115] Prodnik JA . The crisis of legitimacy and the appropriation of resistance in capitalism. tripleC: communication, Capitalism & Critique. Open Access Journal for a Global Sustainable Information Society 2023; 21(1): 51–73.

[116] Wright EO . How to be an anticapitalist in the twenty-first century. Verso Books, 2019.

[117] Teeple G . The democracy that never was: a critique of liberal democracy. Springer Nature, 2024.

[118] Carroll WK Sapinski JP . Organizing the 1%: how corporate power works. Fernwood Publishing, 2018.

[119] Pilon D Savage L . Working-class politics matters: identity, class, parties. In: Albo G Maher S Zuege A (eds). State transformations: Classes, strategy, socialism. Brill, 2021, pp. 76–98.

[120] Coburn D . Income inequality, social cohesion and the health status of populations: the role of neo-liberalism. Soc Sci Med 2000; 51(1): 135–146. 10817476 10.1016/s0277-9536(99)00445-1

[121] Coburn D . Beyond the income inequality hypothesis: class, neo-liberalism, and health inequalities. Soc Sci Med 2004; 58(1): 41–56. 14572920 10.1016/s0277-9536(03)00159-x

[122] Wright EO . The class analysis of poverty. Int J Health Serv 1995; 25(1): 85–100. 7729968 10.2190/WYRM-630N-8M6V-7851

[123] Holstrom N . Exploitation. Can J Philos 1977; 7(2): 353–369.

[124] Øversveen E Kelly CA . Alienation: a useful concept for health inequality research. Scand J Publ Health 2022; 50(7): 1018–1023. 10.1177/14034948221085394 PMC9578084 35549496

[125] Baum F Anaf J Freeman T , et al. Twenty-first century alienation and health: a research agenda. J Epidemiol Community Health 2025; 481–483. 39824546 10.1136/jech-2024-223112

[126] Dawson M . Marxism on Musk: reflections on Baum et al’s ‘Twenty-First Century Alienation and Health’. J Epidemiol Community Health 2025; 79(7): 477–478. 40185615 10.1136/jech-2025-223762

[127] Ross RJ Trachte KC . Global capitalism: the new leviathan. Suny Press, 1990.

[128] Hallstrom L . The politics and policy design of health and inequity in Canada. In: Raphael D (ed). Social determinants of health: Canadian perspectives. Canadian Scholars’ Press, 2025, pp. 542–568.

[129] Bruckmeier K . Transformation of society: governance of global crises. In: The anthropocene and its future: The challenges of accelerating social and ecological change. Cham: Springer International Publishing, 2024, pp. 259–293.

[130] Kotarski K . The new era of polycrisis and how to tackle it. Future Europe Journal 2023; 3: 14–25.

[131] Magatti M . Polycrisis as collapse of the ‘universal and homogeneous state’. Philos Soc Critic 2025; 01914537251316825.

[132] Govender P Medvedyuk S Raphael D . 1845 or 2023? Friedrich Engels’s insights into the health effects of Victorian-era and contemporary Canadian capitalism. Sociol Health Illness 2023; 45(8): 1609–1633. 10.1111/1467-9566.13676 37226700

[133] Woolhandler S Himmelstein DU Ahmed S , et al. Public policy and health in the Trump era. Lancet 2021; 397(10275): 705–753. 33581802 10.1016/S0140-6736(20)32545-9

[134] Bambra C Lynch J Smith KE . Pandemic politics: inequality through public policy. In: The unequal pandemic. Policy Press, 2021, pp. 77–98.

[135] Navarro V . The crisis of the western system of medicine in contemporary capitalism. Int J Health Serv 1978; 8(2): 179–211. 640764 10.2190/JYEY-AND8-XWU4-NR74

[136] Hermann C . The critique of commodification: contours of a post-capitalist society. Oxford University Press, 2021.

[137] Organization for Economic Cooperation and Development . Health spending. 2023. https://www.oecd.org/en/data/indicators/health-spending.html

[138] Ramsey SD Bansal A Fedorenko CR , et al. Financial insolvency as a risk factor for early mortality among patients with cancer. J Clin Oncol 2016; 34(9): 980–986. 26811521 10.1200/JCO.2015.64.6620 PMC4933128

[139] Armstrong P Armstrong H . How privatization infects the Canadian health care system. New Labor Forum 2023; 32: 42–49.

[140] Goodair B Reeves A . The effect of health-care privatisation on the quality of care. Lancet Public Health 2024; 9(3): e199–e206. 38429019 10.1016/S2468-2667(24)00003-3

[141] Goodair B Reeves A . Outsourcing health-care services to the private sector and treatable mortality rates in England, 2013–20: an observational study of NHS privatisation. Lancet Public Health 2022; 7(7): e638–e646. 35779546 10.1016/S2468-2667(22)00133-5 PMC10932752

[142] Lee SK Rowe BH Mahl SK . Increased private healthcare for Canada: is that the right solution? Healthc Policy 2021; 16(3): 30–42. 33720822 10.12927/hcpol.2021.26435 PMC7957357

[143] Zeira A . Mental health challenges related to neoliberal capitalism in the United States. Community Ment Health J 2022; 58(2): 205–212. 34032963 10.1007/s10597-021-00840-7 PMC8145185

[144] Engels F . The condition of the working class in England. Penguin Classics, 1845/2009.

[145] Medvedyuk S Govender P Raphael D . The reemergence of Engels’ concept of social murder in response to growing social and health inequalities. Soc Sci Med 2021; 289: 114377. 34662784 10.1016/j.socscimed.2021.114377

[146] Govender P Medvedyuk S Raphael D . Mainstream news media’s engagement with Friedrich Engels’s concept of social murder. TripleC: communication, Capitalism & Critique. Open Access Journal for a Global Sustainable Information Society 2022; 20(1): 62–81.

[147] Jackson Sow M . Social murder. 2025. https://papers.ssrn.com/sol3/papers.cfm?abstract_id=5142005

[148] Bohrer A . Intersectionality and Marxism: a critical historiography. Hist Mater 2018; 26(2): 46–74.

[149] Yeh ET Bryan J . Indigeneity. In: Perreault T Bridge G McCarthy J (eds). The Routledge handbook of political ecology. Routledge, 2015, pp. 531–544.

[150] Blaut JM . Colonialism and the rise of capitalism. Sci Soc 1989; 53(3): 260–296.

[151] Crook M Short D South N . Ecocide, genocide, capitalism and colonialism: consequences for Indigenous peoples and glocal ecosystems environments. Theor Criminol 2018; 22(3): 298–317.

[152] Radcliffe SA . Geography and indigeneity III: Co-articulation of colonialism and capitalism in indigeneity’s economies. Prog Hum Geogr 2020; 44(2): 374–388.

[153] Medvedyuk S Raphael D . Historical perspectives. In: Bryant T (ed). Handbook on the Social Determinants of Health. Edward Elgar Publishing, 2025, pp. 13–26.

[154] Freudenberg N . Lethal but legal: corporations, consumption, and protecting public health. Oxford, UK: Oxford University Press, 2014.

[155] Lee K Freudenberg N . Public health roles in addressing commercial determinants of health. Annu Rev Publ Health 2022; 43(1): 375–395. 10.1146/annurev-publhealth-052220-020447 34982584

[156] Raphael D . Beyond policy analysis: the raw politics behind opposition to healthy public policy. Health Promot Int 2015; 30(2): 380–396. 24870808 10.1093/heapro/dau044

[157] Scambler G . Capitalists, workers and health: illness as a ‘side-effect’of profit-making. Soc Theor Health 2009; 7: 117–128.

[158] Holden C Lee K . Corporate power and social policy: the political economy of the transnational tobacco companies. Glob Soc Policy 2009; 9(3): 328–354. 20228951 10.1177/1468018109343638 PMC2836532

[159] Smith KE Savell E Gilmore A . What is known about tobacco industry efforts to influence tobacco tax? A systematic review of empirical studies. Tob Control 2013; 22(2): 144–153. 22887175 10.1136/tobaccocontrol-2011-050098 PMC3701860

[160] Langille D . Follow the money: how business and politics define our health. In: Raphael D (ed). Social determinants of health: Canadian perspectives. Canadian Scholars’ Press, 2025, pp. 481–503.

[161] Drache D Froese MD . Has populism won? The war on liberal democracy. ECW Press, 2022.

[162] Schmidt I . The populist race: neoliberalism falling behind, new right forging ahead, the left stumbling along. Perspect Global Dev Technol 2019; 18(1-2): 61–78.

[163] Raphael D Bryant T . Politics, policies, practices and outcomes: despite Canada’s reputation, the nordic nations are the leaders in health promotion. Soc-Med Tidskr 2020; 97(3): 373–392.

[164] Rinaldi C Bekker MP . A scoping review of populist radical right parties’ influence on welfare policy and its implications for population health in Europe. Int J Health Pol Manag 2020; 10(3): 141. 10.34172/ijhpm.2020.48 PMC7947904 32610727

[165] Stronks K Agyemang C . “The multifaceted pathways linking populism to ethnic minority. Health comment on” a scoping review of populist radical right parties’ influence on welfare policy and its implications for population health in Europe. Int J Health Pol Manag 2020; 10(9): 588–590. 10.34172/ijhpm.2020.154 PMC9278369 32823378

[166] Bryant T Raphael D . The old mole and the new democratic party: why the NDP is an impediment to social progress in Canada. Social Democr 2023; 37(3): 139–166.

[167] Winlow S Hall S . The death of the left: why we must begin from the beginning again. Policy Press, 2022.

[168] Manwaring R . The politics of social democracy: issues, dilemmas, and future directions for the centre-left. Routledge, 2021.

[169] Selby J . Labour in need of revolutionary vision. Journal of Canadian Labour Studies 2019; 83: 233–246.

[170] Przeworski A . How many ways can be third? In: Glyn A (ed). Social democracy in neoliberal times. Oxford University Press, 2001, pp. 312–333.

[171] Farris SR Horton A Lloyd E . Corporatisation and financialisation of social reproduction: care homes and childcare in the United Kingdom. Environ Plann 2024.

[172] Duina F . The Great Breach: populism and the undermining of the public sphere with the logic of the private one. Populism 2024; 7(2): 174–196.

[173] Hartwich L Becker JC . Exposure to neoliberalism increases resentment of the elite via feelings of anomie and negative psychological reactions. J Soc Issues 2019; 75(1): 113–133.

[174] Bambra C Lynch J Smith KE . The unequal pandemic: COVID-19 and health inequalities. Policy Press, 2021.

[175] Bryant T Aquanno S Raphael D . Unequal impact of COVID-19: emergency neoliberalism and welfare policy in Canada. Crit Stud 2020; 15(1): 22–39.

[176] Himmelstein DU Woolhandler S . Privatization in a publicly funded health care system: the US experience. Int J Health Serv 2008; 38(3): 407–419. 18724573 10.2190/HS.38.3.a

[177] Woolhandler S Himmelstein D . Healthcare reform 2.0. Soc Res: Int Q 2011; 78(3): 719–730.

[178] Whiteside H McBride S Evans B . Transforming the public sector. In: Varieties of austerity. Bristol University Press, 2021, pp. 81–102.

[179] Lynch J . Regimes of inequality: the political economy of health and wealth. Cambridge University Press, 2020.

[180] Curry-Stevens A Raphael D . Surmounting the barriers: making action on the social determinants of health a public policy priority. In: Raphael D (ed). Social determinants of health: Canadian perspectives. Canadian Scholars’ Press, 2025, pp. 619–647.

[181] Raphael D Bryant T Mikkonen J , et al. The Canadian facts. Ontario Tech University and York University, 2020. https://thecanadianfacts.org/

[182] Esping-Andersen G . The three worlds of welfare capitalism. Princeton University Press, 1990.

[183] Esping-Andersen G . Social foundations of post-industrial economies. Oxford University Press, 1999.

[184] Pontusson J . Inequality and prosperity: social Europe vs. liberal America. Cornell University Press, 2005.

[185] Esping-Andersen G . Politics against markets: the social democratic road to power. Princeton University Press, 2085.

[186] Rostila M . Gøsta Esping-Andersen: welfare regimes and social inequalities in health. In: Locock L Ziebland S Collyer F (eds). The Palgrave handbook of social theory in health, illness and medicine. London: Palgrave Macmillan UK, 2015, pp. 644–659.

[187] Conference Board of Canada . Society. 2017. https://www.conferenceboard.ca/hcp/society-aspx/

[188] Hellmann T Schmidt P Heller M . Social justice in the EU and OECD index report 2019. Bertelsmann Stiftung, 2019. https://www.bertelsmann-stiftung.de/en/publications/publication/did/social-justice-in-the-eu-and-oecd

[189] Bambra C . Going beyond the three worlds of welfare capitalism: regime theory and public health research. J Epidemiol Community Health 2007; 61(12): 1098–1102. 18000134 10.1136/jech.2007.064295 PMC2465657

[190] Bambra C . Health inequalities and welfare state regimes: theoretical insights on a public health ‘puzzle’. J Epidemiol Community Health 2011; 65(9): 740–745. 21690243 10.1136/jech.2011.136333

[191] Bambra C . Social inequalities in health: the Nordic welfare state in a comparative context. Changing social equality: the Nordic welfare model in the 21st century. Bristol University Press, 2012, pp. 143–164.

[192] Bambra C . States of health: welfare regimes, health, and healthcare. In: The Routledge handbook of the welfare state. Routledge, 2012, pp. 282–295.

[193] Bambra C . In defence of (social) democracy: on health inequalities and the welfare state. J Epidemiol Community Health 2013; 67(9): 713–714. 23814270 10.1136/jech-2013-202937

[194] Brennenstuhl S Quesnel-Vallée A McDonough P . Welfare regimes, population health and health inequalities: a research synthesis. J Epidemiol Community Health 2012; 66(5): 397–409. 22080814 10.1136/jech-2011-200277

[195] Muntaner C Borrell C Ng E , et al. Politics, welfare regimes, and population health: controversies and evidence. Sociol Health Illness 2011; 33(6): 946–964. 10.1111/j.1467-9566.2011.01339.x 21899562

[196] McCartney G Hearty W Arnot J , et al. Impact of political economy on population health: a systematic review of reviews. Am J Publ Health 2019; 109(6): e1–e12. 10.2105/AJPH.2019.305001 PMC6507992 31067117

[197] Organization for Economic Cooperation and Development OECD . Infant mortality. 2025. https://www.oecd.org/en/data/indicators/infant-mortality-rates.html

[198] Berkowitz SA . Health Keynesianism: why full employment policy matters for population health. J Gen Intern Med 2024; 39(10): 1914–1916. 38600402 10.1007/s11606-024-08756-0 PMC11282018

[199] Berkowitz SA . Equal care: health equity, social democracy, and the egalitarian state. JHU Press, 2024.

[200] Raphael D . An analysis of international experiences in tackling health inequalities. In: Raphael D (ed). Tackling health inequalities: Lessons from international experiences. Canadian Scholars’ Press, 2012, pp. 229–264.

[201] LeysHealth C. health care and capitalism. Social Regist 2010; 46: 1–28.

[202] Mitchell J . What is to be done about health and illness? Penguin, 1984.

[203] Navarro V . Has socialism failed? An analysis of health indicators under socialism. Int J Health Serv 1992; 22(4): 583–601. 1399170 10.2190/B2TP-3R5M-Q7UP-DUA2

[204] Waitzkin H . A Marxist view of medical care. Ann Intern Med 1978; 89(2): 264–278. 354452 10.7326/0003-4819-89-2-264

[205] Waitzkin H . The social origins of illness: a neglected history. Int J Health Serv 1981; 11(1): 77–103. 7016768 10.2190/5CDV-P4FE-Y6HN-JACD

[206] Waitzkin H . The second sickness: contradictions of capitalist health care. Rowman & Littlefield Publishers, 2000.

[207] Waitzkin H . Moving beyond capitalism for our health. Int J Health Serv 2020; 50(4): 458–462. 32370687 10.1177/0020731420922827

[208] Waitzkin H (ed). Health care under the knife: Moving beyond capitalism for our health. NYU Press, 2018.

[209] Carroll WK . Conclusion. In: Carroll W (ed). Regime of obstruction: How corporate power blocks energy democracy. Athabasca University Press, 2021, pp. 479–504.

[210] Benedetto G Hix S Mastrorocco N . The rise and fall of social democracy, 1918-2017. Am Polit Sci Rev 2020; 114(3): 928–939.

[211] Keating M (ed). Crisis of social democracy in Europe. Edinburgh University Press, 2013.

[212] Polacko M . The rightward shift and electoral decline of social democratic parties under increasing inequality. West Eur Polit 2022; 45(4): 665–692.

[213] Evans B Schmidt I (eds). Social democracy after the cold war. Athabasca University Press, 2012.

[214] Vike H Fagertun A Haukelien H . Introduction: welfare state capitalism, universalism, and social reproduction in Scandinavia. The political economy of care: Welfare state capitalism, universalism, and social reproduction. Scandinavian University Press, 2024, pp. 9–40.

[215] Shammas VL . The global hinterland of social democracy: on the limitations of Norwegian welfare capitalism. Nordisk välfärdsforskning 2024; 9: 66–83.

[216] Miéville C . A spectre, haunting: on the communist manifesto. Head of Zeus, 2021.

[217] Harvey D . Seventeen contradictions and the end of capitalism. Oxford University Press, 2014.

[218] Kovel J . Ecosocialism, global justice, and climate change. Capitalism Nature Socialism 2008; 19(2): 4–14.

[219] Flanagan E. Raphael D. Canadian credit unions and the prospects for a post-capitalist economy. Cap Cl. 2025.

